# Biosynthesis of Bt-Ag_2_O nanoparticles using *Bacillus thuringiensis* and their pesticidal and antimicrobial activities

**DOI:** 10.1007/s00253-023-12859-9

**Published:** 2024-01-22

**Authors:** Jiajia Ge, Jianzhong Hu, Sufen Cui, Yirong Wang, Caijiayi Xu, Wenzhuo Liu

**Affiliations:** School of Grain Science and Technology, Jiangsu University of Science and Technology, Zhenjiang, 212100 China

**Keywords:** Ag_2_O NPs, *Bacillus thuringiensis*, Insecticidal activity, Antifungal activity, Biosynthesis

## Abstract

**Abstract:**

Nanosilver oxide exhibits strong antibacterial and photocatalytic properties and has shown great application potential in food packaging, biochemical fields, and other fields involving diseases and pest control. In this study, Ag_2_O nanoparticles were synthesized using *Bacillus thuringiensis* (Bt-Ag_2_O NPs). The physicochemical characteristics of the Bt-Ag_2_O NPs were analyzed by UV‒vis spectroscopy, Fourier transform infrared spectroscopy (FTIR), X-ray diffraction (XRD), scanning electron microscope (SEM), inductively coupled plasma emission spectrometry (ICP), high-resolution transmission electron microscopy (HR-TEM), and zeta potential. The phis-chemical characterization revealed that the Bt-Ag_2_O NPs are in spherical shape with the small particle size (18.24 nm), high crystallinity, well dispersity, and stability. The biopesticidal and antifungal effects of Bt-Ag_2_O NPs were tested against *Tribolium castaneum*, *Aspergillus flavus*, and *Penicillium chrysogenum*. The survival, growth, and reproduction of tested pests and molds were significantly inhibited by Bt-Ag_2_O NPs in a dose-dependent manner. Bt-Ag_2_O NPs showed higher pesticidal activities against *T. castaneum* than Bt and commercial Ag_2_O NPs. The LC_50_ values of Bt, Ag_2_O NPs, and Bt-Ag_2_O NPs were 0.139%, 0.072%, and 0.06% on day 14, respectively. The Bt-Ag_2_O NPs also showed well antifungal activities against *A. flavus* and *P. chrysogenum*, while it resulted a small inhibition zone than commercial Ag_2_O NPs did. In addition, *A. flavus* showed much more sensitive to Bt-Ag_2_O NP treatments, compared to *P. chrysogenum*. Our results revealed that Bt-Ag_2_O NPs synthesized using *B. thuringiensis* could act as pesticides and antifungal agents in stored-product fields.

**Key points:**

*• Bt-Ag*_*2*_*O NPs could be synthesized using Bacillus thuringiensis (Bt)*.

*• The NPs showed a high degree of crystallinity, spherical shape, and small particle size*.

*• The NPs also showed excellent insecticidal and antifungal activity*.

**Supplementary Information:**

The online version contains supplementary material available at 10.1007/s00253-023-12859-9.

## Introduction

The worldwide population is estimated to reach 10 billion people by 2050, and the total global food demand is expected to increase by 30 to 62% between 2010 and 2050. During the same period, the population at risk of hunger is expected to change by − 91% to + 30%. Approximately 25% of food produced is lost and wasted in all food chains, from field to fork (Van Dijk et al. 2021). The economic cost of postharvest losses is always a risk, and it is not easy to precisely estimate the loss due to pests and fungi (Boyer et al. 2012; Islam 2017). Currently, methods to control these pests involve using fumigants with chemical insecticides, which are generally the most effective management methods. However, when these chemicals are used in the long term, pests may develop resistance and excessive pesticide residues accumulate in treated grains, which may cause human health and environmental problems. Thus, an increasing number of researchers have focused on novel and potentially eco-friendly control methods (Benelli 2018).

Recently, nanotechnology has provided strategies for developing new pesticides. Nanomaterials are ultrafine particle materials with particle sizes of less than 100 nm that exhibit peculiar physicochemical properties due to increased size confinement, surface area, and quantum effects; as a result, nanomaterials are widely used in industry and daily life (Danish et al. 2022). Various nanomaterials, such as Si/SiO_2_, Ag/Ag_2_O, Al_2_O_3_, TiO_2_, and ZnO nanoparticles (NPs), have been investigated for their antibacterial and pesticidal activities in pest control and food storage (Benelli 2018; Dharmaraj et al. 2021; Elbahnasawy et al. 2021; Hossain et al. 2023; Krol et al. 2017; Shehabeldine et al. 2021; Wang et al. 2011). These NPs can effectively control pests, and pests do not easily develop resistance to these materials. Among these NPs, Ag_2_O NPs have recently attracted more attention for application in the fields of sensors, photocatalysts, photovoltaic cells, fuel cells, diagnostic biological probes, optical storage media, food packaging, etc., due to their strong photocatalytic activity, broad-spectrum bacteriostatic activity, ability to absorb light, and oxidation activity (Danish et al. 2022; Patel and Joshi 2023; Shaaban et al. 2023; Wang and Gu 2009). The production of silver-related nanomaterials is expected to increase from 360–450 tons in current years to 825 tons per year by 2025 (Altaf et al. 2021).

The biological method of preparing metallic nanomaterials has become popular because it is cheaper and more ecological than the physiochemical synthesis methods, and the materials are much safer to handle (Ahmed et al. 2017; Shehabeldine et al. 2021). Various of microbial agents, such as bacteria (i.e., *Lactobacillus sporogens*, *Aeromonas hydrophila*, and *Bacillus cereus*), fungi (i.e., *Volvariella volvacea*, *Phanerochaete chrysosporium*, and *Xylaria arbuscula*), and yeast (i.e., *Lactobacillus* sp. and *Papiliotrema laurentii*), have been reported to be effective for NP synthesis (Danish et al. 2022; Devi et al. 2023; Jadoun et al. 2022; Krol et al. 2017; Nehru et al. 2023; Majumder et al. 2023; Malaikozhundan et al. 2017). These microbes could produce NPs intracellularly or extracellularly, and the process of intracellular synthesis is more complicated than that of extracellular synthesis due to the greater number of organelles inside the microbial cell. During the process of NP synthesis, the enzymes and phytochemicals from bacterial cells perform the redox reactions with the precursors, leading to the formation of metal/metal oxide nanoparticles. The synthesis of NPs using microbes results from microbial metal ion detoxification, including reduction and/or chemiosmotic cations or proton antitransporters (Dharmaraj et al. 2021; Narayanan and Sakthivel 2010). Recently, a few studies have reported Ag_2_O NPs synthesized using microorganisms, such as *Lactobacillus mindensis*, *Nitrobacter* sp., *Bacillus paramycoides*, cell-free supernatant of *Bacillus thuringiensis* SSV1, and *Xanthomonas* sp. P5 (Danish et al. 2022; Nguyen et al. 2023; Singh et al. 2022). The Ag_2_O NPs synthesized using microbes always showed excellent antibacterial and insecticidal activities as well as good stability (Dharmaraj et al. 2021; Hasanin et al. 2021). These studies mostly highlight photocatalysis, environmental remediation, and biomedical applications, but relatively few studies have been conducted on their applications in grain storage as biopesticides and antifungal agents (Danish et al. 2022; Singh et al. 2018).

*Bacillus thuringiensis* (Bt), a member of the *Bacillus cereus sensu* lato (Bcsl) complex, is currently the most widely used microbial insecticide against insect pests. Various types of Bt produce different kinds of parasporal protein crystals that contain δ-endotoxins, which play an important role in Bt toxicity (Elgizawy and Ashry 2019). In addition, Bt prevents the growth of plant pathogens, such as phytopathogenic *Verticillium* sp. and *Fusarium verticillioides*, because of *bacillus* lipopeptides (i.e., surfactins, iturins, and fengycins) or chitinases (Hollensteiner et al. 2017). However, various insects have developed resistance following the application of Bt production (Wu 2014). To protect and effectively utilize these natural biological insecticides and antifungal agents, the new functions of Bt and their application need to be further developed.

In this study, Ag_2_O NPs were first synthesized using *B. thuringiensis* (Bt-Ag_2_O NPs), and then, their physical structure and chemical composition were characterized using modern analytical techniques, such as X-ray diffraction (XRD), UV‒vis spectroscopy (UV‒vis), Fourier transform infrared spectroscopy (FTIR), scanning electron microscopy (SEM), high-resolution transmission electron microscopy (HR-TEM), and inductively coupled plasma emission spectrometry (ICP) analysis. The pesticidal and antifungal activities of Bt-Ag_2_O NPs against *Tribolium castaneum* Herbst (a typical stored product insect pest), *Aspergillus flavus*, and *Penicillium chrysogenum* (two typical cereal microorganisms) were investigated.

## Materials and methods

### Materials

Luria–Bertani (LB) agar was prepared with 1% tryptone and 0.5% yeast extract and then sterilized. Tryptone and yeast extract were purchased from McLean Biochemical Technology Co., Ltd., Shanghai, China; Potato Dextrose Agar (PDA) was purchased from Bioway Technology Co., Ltd., Shanghai, China; 0.1 mol/L silver nitrate solution and Ag_2_O NPs (Cas No. 20667–12-3, 20 nm) were purchased from Ji zhi Biochemical Technology Co., Ltd., Shanghai, China; all aqueous solutions were prepared from deionized water.

### Insect rearing

*T. castaneum* Herbst was cultivated in the insect breeding room of the School of Grain Science of Jiangsu University of Science and Technology. The insects were fed with whole wheat flour containing 5% yeast (WWF) in an environmentally controlled chamber at 28 ± 2 °C and 65 ± 5% R.H.

### Bacterial and fungal culture

Gram-positive *B. thuringiensis* (ACCC 03343), *A. flavus* (CICC 2472), and *P. chrysogenum* (CICC 4031) were obtained from the China Center of Industrial Culture Collection, Beijing, China. *B. thuringiensis* was inoculated in LB solid medium and incubated at 30 °C for 24 h. *A. flavus* and *P. chrysogenum* were inoculated on PDA solid medium and maintained at 28 °C for subsequent experiments.

### Bt-Ag_2_O NPs synthesized using B. thuringiensis

The synthesis was based on a method described by Malaikozhundan with slight modifications (Karunagaran et al. 2017; Malaikozhundan et al. 2017). Under sterile conditions, *B. thuringiensis* was inoculated into a 500-mL flask containing 100-mL LB liquid medium and incubated in an orbital shaker at 30 °C and 150 rpm for 24 h. It was then diluted 4 times and cultivated for another 24 h. After incubation, we adjusted the pH to 7.5 using 0.4 M NaOH solution, added 100 mL 0.1 M silver nitrate solution into the *B. thuringiensis* solution, and transferred it into a water bath at 75 °C for 15 min. When the reaction was complete, we transferred the flask into an incubator at 30 °C for 12 h. The precipitates were then obtained after filtration, washed with distilled water, and dried in a hot air oven at 40 °C for 4 h.

### Characterization of synthesized Bt-Ag_2_O NPs

#### UV‒vis spectral analysis

The Bt-Ag_2_O NPs synthesized using *B. thuringiensis* were measured using a UV‒vis spectrophotometer (UV‒vis spec. Shimadzu 1601, Japan) in the range of 200 to 800 nm, and the maximum absorbance (*λ*_max_) was recorded (Barbhuiya et al. 2022).

#### Fourier transform infrared spectroscopy (FTIR) analysis

FTIR analysis of the synthesized Bt-Ag_2_O NPs was carried out using a Fourier transform infrared spectrometer (Thermo, Scientific Nicolet iS20, USA) at a resolution of 2 cm^−1^ within a range of 4000–400 cm^−1^.

#### X-ray diffraction (XRD) analysis

The crystallinity and particle size were analyzed using an X-ray diffractometer (XRD) (Rigaku, Japan) operating at a voltage of 40 kV and a current of 40 mA with Cu-Kα rays. The average size of the particles was determined using Debye Scherrer’s Eq. (1).

1 $$D=\frac{0.94\lambda }{\beta\;cos\;\theta }$$

where *λ* is the wavelength (Cu-Kα), *β* is the full width half-maximum (FWHM) of the Ag_2_O (111) line, and *θ* is the diffraction angle (Malaikozhundan et al. 2017).

#### Scanning electron microscopy (SEM) and energy dispersive spectrometer (EDS) analysis

Scanning electron microscopy (SEM, Hitachi Regulus 8100, Japan) was performed with an accelerating voltage of 3 kV. Energy dispersive spectrometry (EDS) with an accelerating voltage of 20 kV was used to analyze the elemental composition of the nanoparticles.

#### High-resolution transmission electron microscopy(HR-TEM) analysis

Samples (2 mg) were placed in ethanol solution, followed by ultrasonication. Then, 25 μL of sample was sputter-coated on a copper stub, and the images of NPs were studied using high-resolution transmission electron microscopy (HR-TEM, JEM 2100 plus, JEOL Ltd., USA) operating at an accelerating voltage of 200 kV.

#### Zeta potential distribution

The surface charges of the synthesized Bt-Ag_2_O NPs were determined using a zeta potential analyzer (Malvern Zetasizer Nano ZS90, Malvern Instruments LTD., UK).

#### Inductively coupled plasma emission spectrometer (ICP) analysis

The amount of Ag in the synthesized Bt-Ag_2_O NPs was determined following a previous study with slight modification (Liu et al. 2016). A total of 235 mg of sample was added to 6 mL HNO_3_, and after 1 mL H_2_O_2_ was added, the solution was covered and heated at 250 °C for sufficient Ag dissolution. Approximately 2 h later, the sample was uncovered and continuously heated until the bean size solution was left, diluted with deionized water to 25 mL, and centrifuged to obtain the supernatant for future determination using an inductively coupled plasma atomic emission spectrometer (ICP) (Thermos ICP PR0, USA).

The concentration of the analyzed ion was calculated according to Eq. (2).

2 $${C}_{x}=\frac{{C}_{0}\times f\times {V}_{0}\times {10}^{-3}}{{C}_{0}\times {10}^{-3}}$$

where *C*_*x*_ is the concentration of the analyzed ion (mg/kg), *c*_0_ is the sample quality (g), *f* is the dilution rate, and *V*_0_ is the final solution after clean up; and Eq. (3).

3 $$W\left(\mathrm{\%}\right)=\frac{{C}_{x}}{{10}^{6}}\times 100\mathrm{\%}$$

where *W* is the content of the analyzed ion (%) and *C*_*x*_ is the same as above.

### Bioactivity of synthesized Bt-Ag_2_O NPs

#### Insecticidal assays

One hundred 2-day-old *T. castaneum* adults were oviposited for 6 h at 28 ± 2 °C and 65 ± 5% R.H. Twenty days later, larvae were used for the following study. The larvicidal activity of Bt-Ag_2_O, Bt, and Ag_2_O NPs was evaluated at five doses of 0.02%, 0.04%, 0.06%, 0.08%, and 0.10%. A total of 20 larvae were selected and placed in a flask containing artificial feed with Bt-Ag_2_O, Bt, or Ag_2_O NPs. Meanwhile, the control experiment was performed with WWF only. Then, the flasks were covered with nylon gauze to allow atmospheric diffusion. All bioassays were performed at 28 ± 2 °C and 65 ± 5% RH in a climate chamber. For each treatment, three replicates were performed. The treatments and control insects were examined every day until the last death, and larval development, pupae, newly emerged adults, and mortality data were recorded.

#### Antifungal assays

The antifungal activities of synthesized Bt-Ag_2_O NPs and Ag_2_O NPs were assessed using the agar well diffusion method. Spore suspensions of *A. flavus* and *P. chrysogenum* were prepared, and the spore number was adjusted to 5 × 10^6^ CFU/mL with microscopy and incubated at 28 °C for 24 h. Stock solutions of Bt-Ag_2_O NPs and Ag_2_O NPs were prepared at different concentrations of 6%, 8%, 10%, 12%, and 14%. PDA solid medium was prepared, and 100 μL of fungal suspension was swabbed uniformly onto PDA medium. Wells were cut out of agar pales using a standard cork borer (5-mm diameter). Then, 100 μL of these stock solutions was added to these wells and inoculated at 28 °C. Seven days later, the inhibition zones were measured for Bt-Ag_2_O NPs and Ag_2_O NPs. All treatments were performed in triplicate.

### Statistical analysis

The mortality of insects in the treatments was analyzed according to the Abbott (1925) equation, as follows:

$$\times\;=\;\frac{\mathrm{mortality\;in\;treatments\;}\left(\mathrm{\;\%}\right)-\mathrm{mortality\;in\;control}(\mathrm{\;\%})}{100-\mathrm{mortality\;in\;control\;}(\mathrm{\;\%})}\times\;100\mathrm{\;\%}$$

where *x* is the actual morality in the treatments. The values of lethal concentration to 50% of insects (LC_50_) were obtained using probit analysis. Data analysis was performed using SPSS for Windows (Version 20.0; IBM Corp., Armonk, NY, USA). Data were analyzed using Student’s test or nonparametric statistical methods followed by the Kruskal‒Wallis test. The results are expressed as the mean ± standard deviation (SD) and were considered statistically significant at *p* < 0.05.

## Results

### Synthesis of Bt-Ag_2_O NPs and UV‒vis analysis

**Fig. 1:**
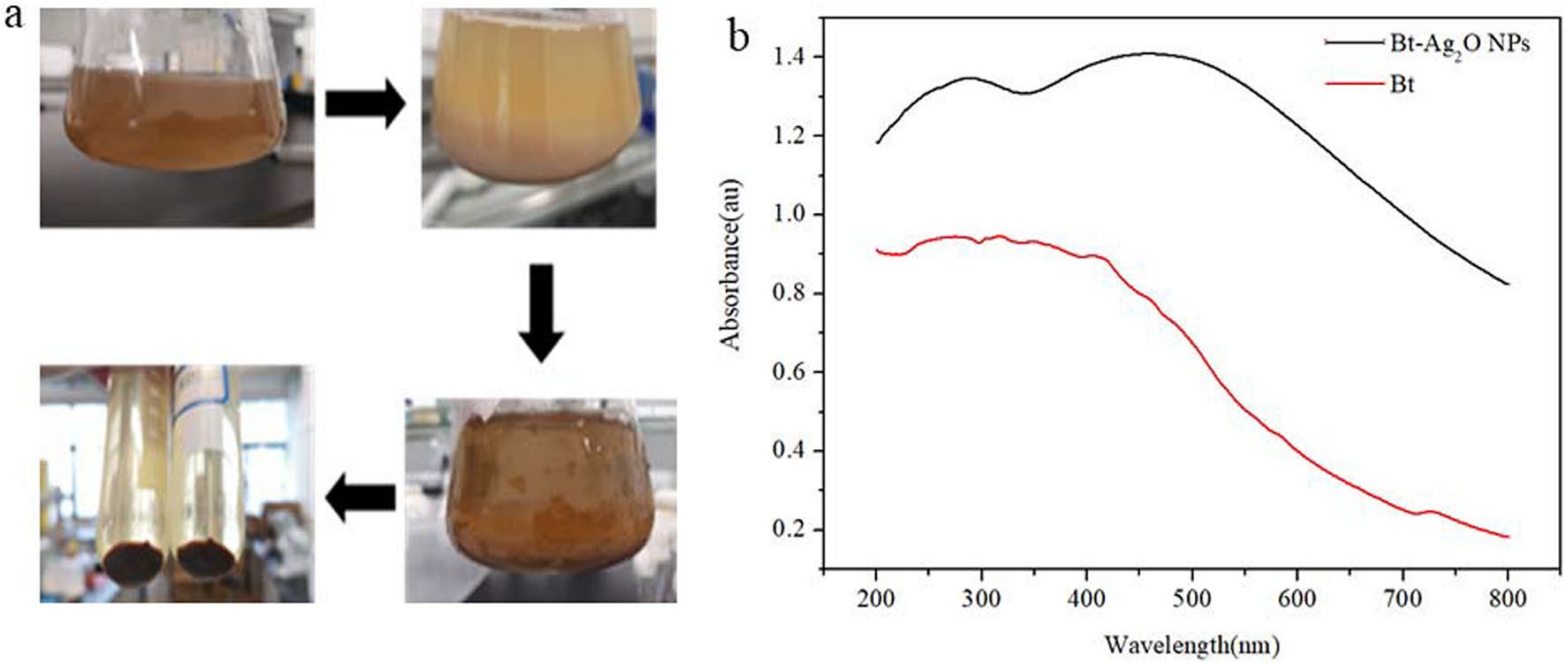
The process (**a**) and UV–vis spectra of *B. thuringiensis* before and after synthesis of Bt-Ag_2_O NPs (**b**)

The synthesis of Bt-Ag_2_O NPs using *B. thuringiensis* was observed through visual changes in color from yellow to brown (Fig. 1a). *B. thuringiensis* solution was brown but clear and then changed to light brown immediately after AgNO_3_ solution was added. The solution changed slowly to dark brown during incubation in a water bath at 75 °C; 25 min later, the reaction was complete, and dark brown precipitates were obtained after filtering. The dried precipitate was ground and stored at − 20 °C for subsequent characterization. UV‒vis spectroscopy was used for this primary characterization of the synthesized NPs. The absorption spectrum of the reaction mixture at different wavelengths ranging from 200 to 800 nm revealed a peak at 460 nm, compared to the control (BT) (Fig. 1b).

### FTIR analysis

**Fig. 2:**
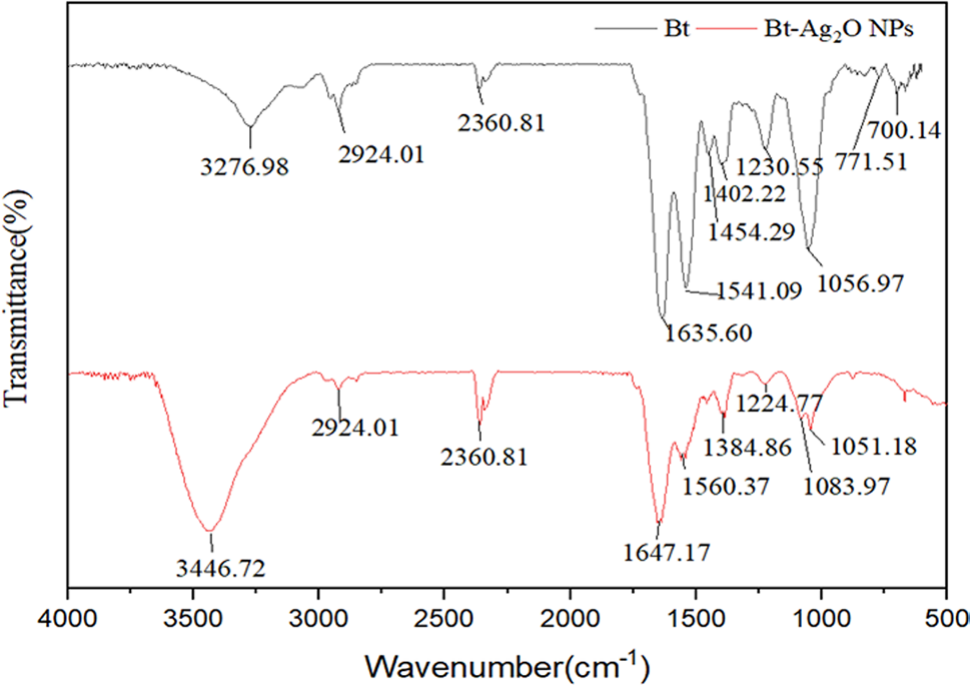
FTIR spectra of *B. thuringiensis* cell before and after synthesis of Bt-Ag_2_O NPs

The functional groups of the synthesized Bt-Ag_2_O NPs and the control (Bt) were characterized by FTIR analysis in the spectral range of 500 to 4000 cm^−1^ (Fig. 2). As shown, prominent peaks were observed at 3276.98 and 3446.72 corresponding to O–H stretching, and the bands at 2924.01 might be attributed to C-H stretching vibrations of alkane; the bands at 2360.81 indicate the presence of C≡C stretching; the bands at 1635.60 and 1647.17 cm^−1^ could correspond to the C = C stretching vibrations of olefin; the peaks at 1541.09 and 1560.37 cm^−1^ correspond to C = C aromatic stretching frequencies; the bands at 1454.29, 1402.22, and 1384.36 cm^−1^ indicated the presence of C-H banding vibrations; the peaks observed at 1051.18–1230.55 cm^−1^ can be endorsed to the C-O and C-N stretching vibrations. The peaks at 700.14 and 771.51 cm^−1^ correspond to the stretching vibration of C-H aromatic groups. The characteristic bands were observed changes in intensities compared to those of *B. thuringiensis* cell before Bt-Ag_2_O NP synthesis.

### XRD pattern analysis

**Fig. 3:**
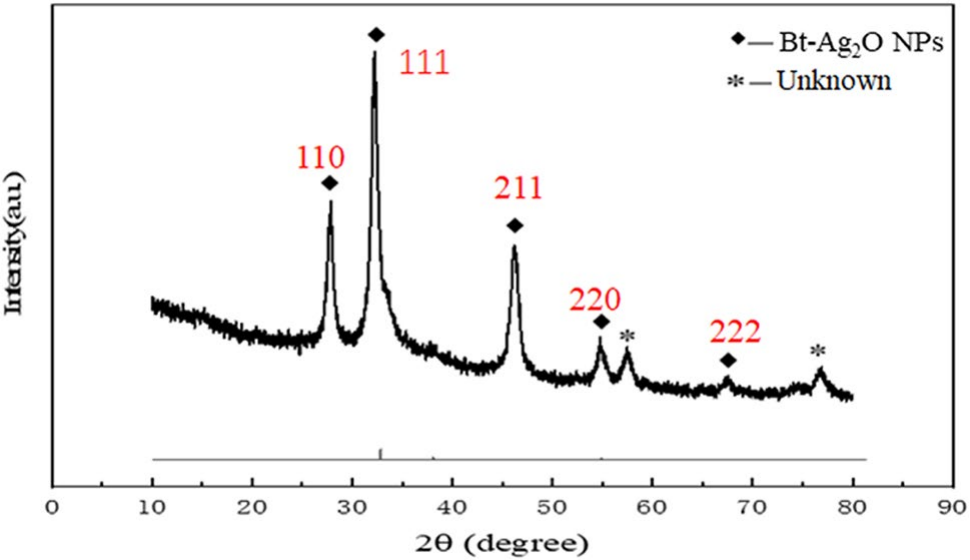
XRD patterns of Bt-Ag_2_O NPs

The nanocrystalline structures of the synthesized Bt-Ag_2_O NPs were determined by XRD (Fig. 3). X-ray diffraction showed strong diffraction peaks at 2θ values of 27.80°, 32.20°, 46.20°, 54.72°, 57.54°, 67.59°, and 76.92°; among them, the intense peaks at 2θ values of 27.80°, 32.20°, 46.20°, 54.72°, and 67.59° correspond to the (110), (111), (211), (220), and (222) planes (referenced with standard JCPDF data file number 43–0997). The average crystalline size of Bt-Ag_2_O NPs was 15 nm using the Debye Scherrer equation.

### SEM and EDS analysis

**Fig. 4:**
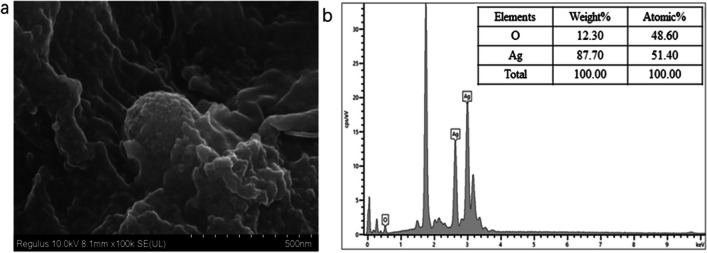
SEM (**a**) and EDS spectra (**b**) of Bt-Ag_2_O NPs

SEM was used to further characterize the morphological structure and size of the synthesized Bt-Ag_2_O NPs (Fig. 4a). The particles were spherical in shape, agglomerated but clearly circular crystals. Energy dispersive spectrometry analysis (EDS) showed the elemental compositions of the NPs. The content ratio of Ag and O was 88.80:11.20 (Fig. 4b).

### HR-TEM analysis

**Fig. 5:**
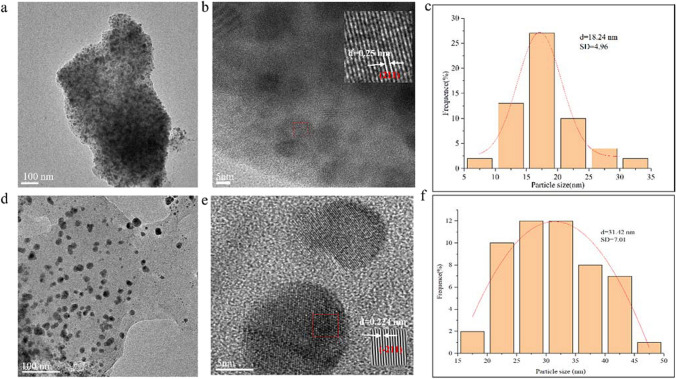
HR-TEM images and particle size distribution histograms of Bt-Ag_2_O NPs. **a**–**c** The original Bt-Ag_2_O NPs. **d**–**f** Bt-Ag_2_O NPs after storage at − 20 °C for 16 months

The shape and size of the synthesized Bt-Ag_2_O NPs were further characterized using HR-TEM (Fig. 5). Bt-Ag_2_O NPs were spherical in shape, well dispersed, and crystalline; the spacing of the lattice fringe is 0.25 nm; the particle sizes of the Bt-Ag_2_O NPs obtained were in the range of 5 to 35 nm, with an average size of 18.24 nm (Fig. 5a–c). Comparing new with stored Bt-Ag_2_O NPs showed no obvious changes in shape, dispersity, and crystallinity, but the average size distribution after storage at − 20 °C for 16 months became bigger (mean diameter 31.42 nm) (Fig. 5d–f).

### Zeta potential analysis

**Fig. 6:**
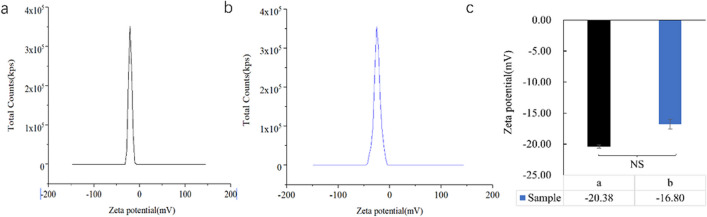
Zeta potential of Bt-Ag_2_O NPs. **a** The original Bt-Ag_2_O NPs. **b** Bt-Ag_2_O NPs after storage at − 20 °C for 16 months. **c** The difference in zeta potential between **a** and **b** samples. Data are expressed as mean ± SD (*n* = 3). Bars labeled by NS represent no statistical significance at *p* < 0.05 using Student’s test

The synthesized Bt-Ag_2_O NPs were negatively charged, and their zeta potential value was − 20.38 mV, and 16 months later, the average zeta potential value became − 16.80 mV (Fig. 6). There were no significant changes in the zeta potential values of the sample after stored for 16 months (*p* < 0.05).

### ICP-OES analysis

Using the ICP-OES test, the content of Ag in the Bt-Ag_2_O NP solution was 3.368 mg/L, and the amount of Ag in the sample was 17.9%, following Eqs. (2) and (3) (Table S1 and Fig. S1).

### Effect of Bt-Ag_2_O NPs on the mortality of *T.* castaneum larvae

**Fig. 7:**
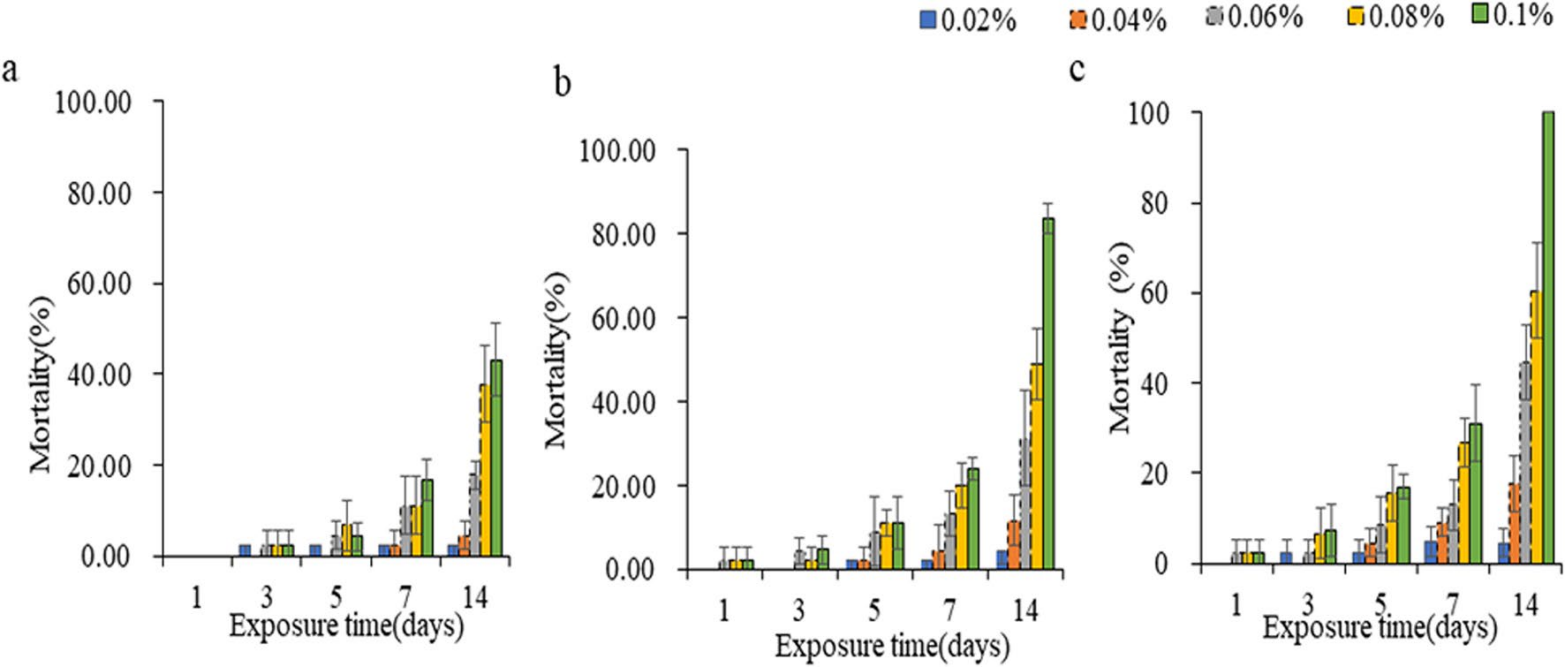
Mortality of *T. castaneum* larvae under Bt (**a**), Ag_2_O NPs (**b**), and Bt-Ag_2_O NPs (**c**) treatment conditions. Data are expressed as mean ± SD (*n* = 3)

The effects caused by Bt, Ag_2_O, and Bt-Ag_2_O NPs at different concentrations on the mortality of *T. castaneum* larvae were assessed (Fig. 7). The mortality of *T. castaneum* larvae increased with increasing Bt, Ag_2_O, and Bt-Ag_2_O NP concentrations and exposure times. Exposure for 14 days to 0.1% Bt-Ag_2_O NPs led to 100% death of larvae, while significant portions of larvae were alive under Bt or Ag_2_O NP treatment conditions. Bt-Ag_2_O NPs showed higher insecticidal activity than the other two pesticides.

### Effect of Bt-Ag_2_O NPs on the development of *T.* castaneum

**Fig. 8:**
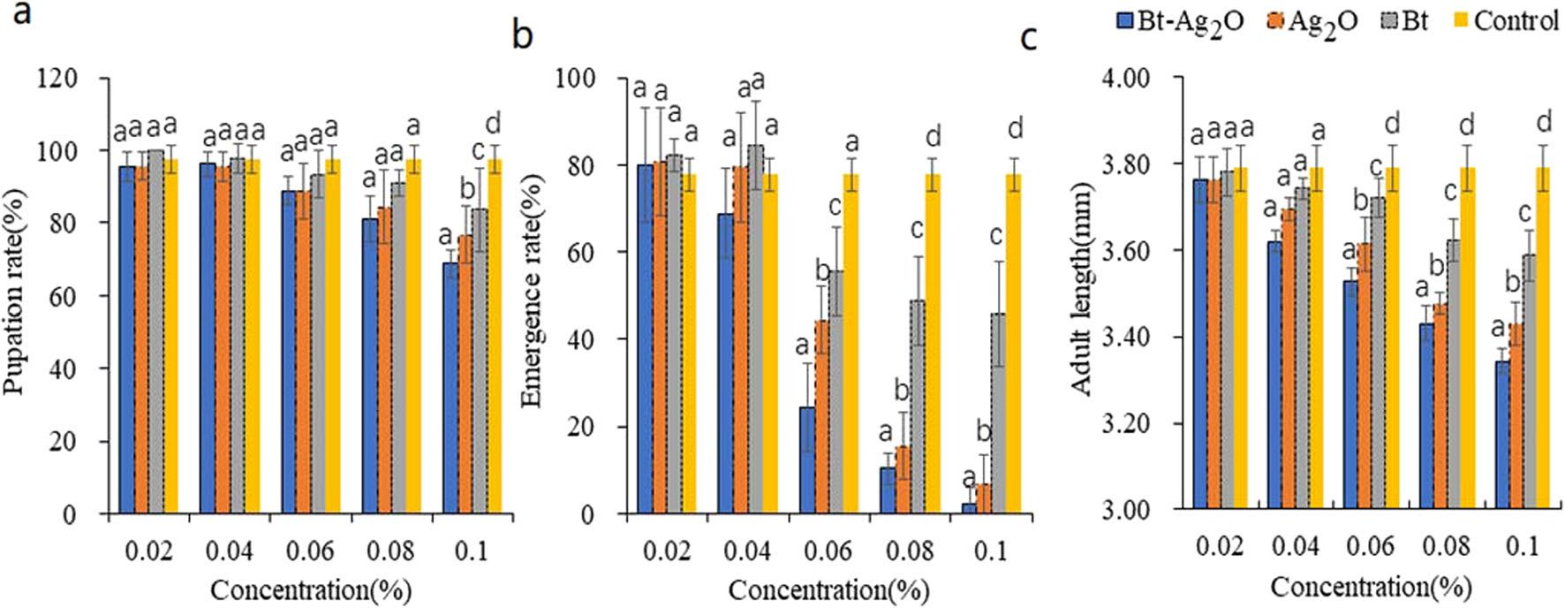
Changes in pupation (**a**), emergence rate (**b**), and newly emergent adult length (**c**) of *T. castaneum* under different treatments. Data are expressed as mean ± SD (*n* = 3). Bars labeled by different letters represent statistical significance at *p* < 0.05 using nonparametric test methods of Kruskal‒Wallis

During the experiment, the surviving insects continued to grow, but the pupation and emergence rates decreased, and the newly emerged adult size decreased compared with that of the controls (Fig. 8). The pupation and emergence rates of *T. castaneum* treated with Bt-Ag_2_O NPs, Ag_2_O NPs, and Bt decreased with increases in the reagent dose, but there were no significant differences in pupation among the three treatments at lower concentrations, and significant differences were only observed among the three groups treated with the three reagents at higher concentrations. The body length of newly emerged adults was also affected by treatments, which was much smaller in treatment with Bt-Ag_2_O NPs, Ag_2_O NPs, and Bt at higher doses than in the controls. At a concentration of 0.1%, the pupation, emergence rate, and newly emerged adult length of *T. castaneum* were significantly different among the three treatments, and the order of pesticidal activities from high to low was Bt-Ag_2_O NPs, Ag_2_O NPs, and Bt.

### Effect of synthesized Bt-Ag_2_O NPs against *A.* flavus and *P.* chrysogenum

**Fig. 9:**
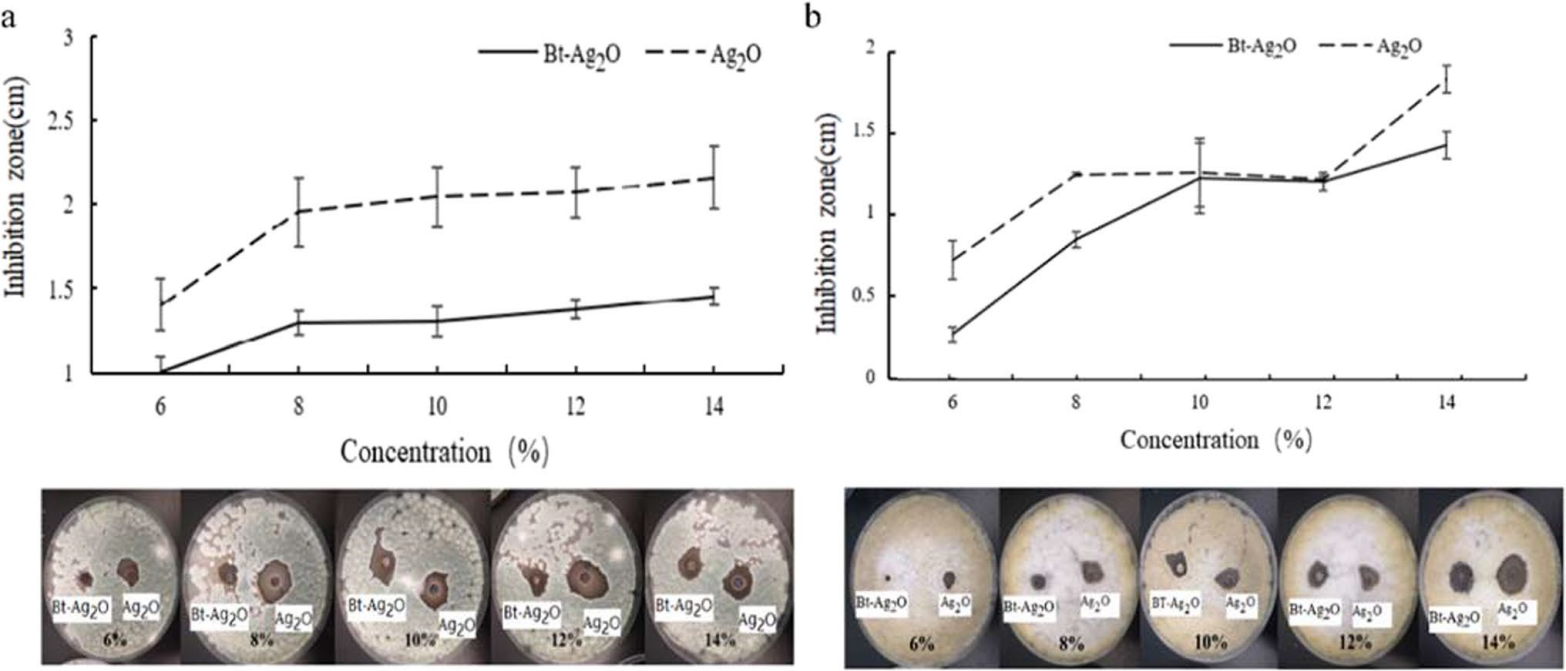
Antifungal activities of Bt-Ag_2_O and Ag_2_O NPs against of *P. chrysogenum* (**a**) and *A. flavus* (**b**)

The antifungal activities of synthesized Bt-Ag_2_O NPs were tested against *P. chrysogenum* and *A. flavus* at different concentrations. The controls were treated with Ag_2_O NPs purchased from a company (Fig. 9). Bt-Ag_2_O NPs and Ag_2_O NPs showed dose-dependent antifungal activities. The inhibition zone of Bt-Ag_2_O NPs observed against *P. flavus* was clearly visible at all concentrations except below 6%, and the zone became larger with increasing concentration. At the same concentration, the inhibition zone diameters observed for Ag_2_O NPs were larger than those observed for Bt-Ag_2_O NPs (Fig. 9a). For example, the inhibition zones of Bt-Ag_2_O and Ag_2_O NPs against *P. chrysogenum* were 1.3 cm and 2.1 cm at 14%, respectively.

Significant antifungal activity was observed against *A. flavus*, and the diameters of the inhibition zones of Bt-Ag_2_O and Ag_2_O NPs increased with increasing concentration, but there was a plateau stage in the concentration range of 10 ~ 12%. Compared with that of Bt-Ag_2_O NPs, the effect of Ag_2_O against *A. flavus* was much stronger in general. The inhibition zones of Bt-Ag_2_O and Ag_2_O NPs against *A. flavus* were 1.45 cm and 1.7 cm at 14%, respectively (Fig. 9b). In addition, *A. flavus* was much more sensitive than *P. chrysogenum* under the same conditions based on the inhibition zone.

## Discussion

*B. thuringiensis* is widely used as an environmental and safe microbial pesticide that can cause harmful effects on nontarget animals. However, as the application of the pesticide has broadened in the agricultural field, insects have developed resistance to Bt and its related products (Wu 2014). Nanometallic materials with particle sizes < 100 nm show unique properties, and some of these materials, such as Ag NPs, Ag_2_O NPs, Al_2_O_3_ NPs, and ZnO NPs, even exhibit great insecticidal and antimicrobial activities (Benelli 2018). Recently, the biosynthesis of nanoparticles using bacteria has shown great potential, as the method is inexpensive and ecological, exhibits high stability, and shows bioactivity (Miu and Dinischiotu 2022). In this study, we synthesized Ag_2_O NPs using *B. thuringiensis* and analyzed their physical and chemical characteristics by different methods (i.e., UV, XRD, zeta potential, ICP, SEM, and TEM). We then applied it against a typical pest (*T. castaneum*) and two fungi (*A. flavus* and *P. chrysogenum*) of stored products to evaluate its pesticidal and antifungal activities.

The biosynthesis procedure was followed by observing visual change in color of the reaction mixture, which turned from yellow to reddish brown (Dhoondia and Chakraborty 2012; Shaaban et al. 2023). A previous study reported that the brown color change resulted from the excitation of surface plasmon resonance vibrations in the metal nanoparticle solution (Yoo et al. 2019). UV‒vis spectroscopy is usually used to determine the optical properties and surface plasmon resonance of nanomaterials. In this study, Bt-Ag_2_O NPs showed a maximum wavelength (*λ*_max_) at 460 nm, which was slightly redshifted compared to previous studies (Dharmaraj et al. 2021). It has been reported that the biosynthesized silver oxide nanoparticles mediated by *Lactobacillus* and *Xanthomonas* sp. P5 had a characteristic peak at 430 nm (Dhoondia and Chakraborty 2012; Krol et al. 2017; Yoo et al. 2019), and the single peak correlating with the *λ*_max_ of the synthesized Ag NPs using *B. thuringiensis* MAE 6 and *Nigrospora oryzae* extracts was at 420 nm (Dawoud et al. 2021; Hashem et al. 2022). These studies revealed that Ag NPs possess unique optical properties, with strongly characteristic surface plasmon resonance (SPR) peaks between 400 and 450 nm, and the SPR sensing indicates the particle size, shape, surrounding, dielectric medium, and the state of aggregation of NPs (Hashem et al. 2022; Patel and Joshi 2023). This is characteristic of the SPR of silver but shows little deviation (Dhoondia and Chakraborty 2012; Krol et al. 2017). The UV‒vis spectrum *λ*_max_ of silver NPs synthesized using *Moringa oleifera* leaf extract was observed at 466 nm (Shaaban et al. 2023).

The changes in Bt-Ag_2_O NPs spectra might result from the out-of-plane quadrupole resonance of the silver nanoparticles, which is reflected in a redshift. This redshift revealed that the NPs obtained were slightly agglomerated and larger than Ag NPs, and this property might be affected by several factors, such as microbial species, pH, incubation temperature, reaction time, and dosage of the precursor substance (Krol et al. 2017). Many mechanisms contribute to metal tolerance in microbes, including bioleaching and bioremediation processes, through Ag hyperaccumulating microbes which can be used as a source of precursors to form silver oxide NPs (Narayanan and Sakthivel 2010). Although many reports have been published, the details remain unclear. Tangential to the relatively smooth absorption curve, the intersection is the band side wavelength. According to the formula *E* = *hv*, the band gap width of the absorption curve was approximately 2.1 eV, which was blue shifted compared with that of the standard silver oxide (2.25 eV). This might result from the interference caused by bacterial metabolites with the characteristic absorption peaks of silver oxide. It should be noted that the peak around 460 nm of synthesized Bt-Ag_2_O NPs is broader than that of chemical synthesized silver-based NPs. It might be because of the mixed morphology or inhomogeneous distribution of the Ag NPs and Ag_2_O NPs (Ashokraja et al. 2017; Yoo et al. 2019). This might be further confirmed by SEM and HR-TEM imaging.

The FTIR spectra of *B. thuringiensis* cell before and after Bt-Ag_2_O NPs synthesized showed slightly changes in intensities of some functional groups such as O–H, C-O, C = C, and C-N groups, similar to the reports about silver oxide NPs synthesized using *Bacillus paramycoides* and *Xanthomonas sp.* P5 (Dharmaraj et al. 2021; Yoo et al. 2019). These functional groups are first associated with Ag ions, and then, the biomolecules of *B. thuringiensis* become part of the nanoparticles after the reaction, acting as a capping agent and making the silver oxide nanoparticles disperse well with good stability (Hasanin et al. 2021). The results further revealed that compounds such as proteins, reducing sugars, and phenolic and aromatic compounds in *B. thuringiensis* could be involved in the synthesis process of Bt-Ag_2_O NPs (Salgado et al. 2023). It has been reported that extracellular hydrogenase, carboxyl groups in tyrosine residues, nitrate reductase, and other peptides/proteins might be responsible for the reduction of Ag ions and the production of silver oxide nanoparticles (Karunagaran et al. 2017).

The XRD pattern of Bt-Ag_2_O NPs showed that peaks were sharpened, indicating the presence of Ag_2_O particles in the particles and their crystalline nature and small size (Singh et al. 2022). These strong diffractions (2θ) at 27.80°, 32.20°, 46.20°, 54.72°, and 67.59° correspond to the (110), (111), (211), (220), and (222) lattice planes of Bragg’s reflection. They were well matched to the face-centered cubic lattices of standard Ag_2_O (PDF 43–0997), thus confirming the formation of Ag_2_O, which could be attributed to spherical nanoparticles (Dhoondia and Chakraborty 2012; Janardhanan et al. 2009; Wei et al. 2011). In the XRD spectra, (110) and (111) are the main indicators of Ag_2_O NPs, and the peaks at (211) and (220) are related to the face-centered cubic crystal structure (Krol et al. 2017; Yoo et al. 2019). Two unknown peaks at 57.54 and 76.92 were also observed, which might result from bacterial precipitation. XRD pattern not only could provide the phase characteristics of crystalline material but also the information about unit cell dimensions (Malaikozhundan et al. 2017).

Particle size and distribution are important for nanoparticles and can directly affect their physical stability, solubility, biocompatibility, and biological activities. These characteristics were determined using XRD and HR-TEM, which revealed that Bt-Ag_2_O NPs were spherical in shape, well dispersed, and crystalline. The average crystalline size of Bt-Ag_2_O NPs was 15 nm using the Debye Scherrer equation, and HR-TEM images showed the sizes of NPs were in the range 5–35 nm with an average size of 18.24 nm. In particular, the average size distributions of Bt-Ag_2_O NPs (mean diameter 31.42 nm) only showed slight changes after storage at − 20 °C for 16 months, which showed the good stability of Bt-Ag_2_O NPs synthesized using *B. thuringiensis*. Organic capping agents increased the stability of the synthesized NPs, which could thus be preserved without precipitation for over a year (Thomas 2017). Such wide range of size distributions might be helpful to the metallic capping efficiencies of functionals groups that both exist in or out of *B. thuringiensis* cell. Similarly, the presence of particles with irregular morphologies in the case of Bt-Ag_2_O NPs could be attributed to the functional groups dependent growth of NPs and leading to the irregular morphologies (Ashokraja et al. 2017). This might be the reason for the broad absorption peak in UV–vis spectra of samples.

The shape of the NPs might affect its biological properties, such as antimicrobial and pesticidal activities. It has been reported that spherical nanoparticles can easily penetrate into the cell and cause death (Sivapriyajothi et al. 2014). Multiple nucleation might occur when precursors are continuously supplied, leading to the inhomogeneity of growth kinetics during nanoparticle synthesis and random condensation (Peralta-Videa et al. 2016). This eventually leads to the formation of polydisperse silver nanoparticles. This aggregate formation revealed the presence of surface-bound biomolecules, including proteins, oligosaccharides, and other metabolites from *B. thuringiensis*; these molecules reduced silver ions to silver or silver oxide nanoparticles and led to changes in biological activities, such as bacteriostatic and insecticidal activities (Narayanan and Sakthivel 2010).

EDS was used to determine the purity and elemental composition of the particles obtained. The EDS spectrum of the NPs obtained generated an optical absorption bank peak at 2.6 and 3.0 keV, which are characteristic of elemental silver and caused by surface plasmon resonance (Parikh et al. 2008). The presence of other element peaks, especially oxygen, at 0.5 keV revealed the metabolites present on the surface of the NPs. The highest peak indicates the element of Si, which emerged because the sample was prepared using water dispersed on a silicon wafer. In addition, Si, Ag, and O were the main elements in this sample. Data obtained from the XRD and EDS spectra show that the synthesized NPs were Ag_2_O NPs rather than Ag NPs. The results coincide with those of other previous studies (Dharmaraj et al. 2021; Miu and Dinischiotu 2022; Narayanan and Sakthivel 2010). The amount of Ag^+^ in the synthesized Bt-Ag_2_O NPs further determined by ICP was 17.9%, revealing that of other inorganic elements was nearly 82.1%. The content of Ag in the sample might directly affect its pesticidal and antifungal activities. In the TEM image, the spacing of the lattice fringe was 0.25 nm, which corresponded with Bragg’s reflection (211) based on the standard spectrum of Ag_2_O (JCPDS 43–0997) in the XRD spectra. The results clearly revealed the high crystallinity and face-centered cubic (FCC) crystal structure of the particles.

The zeta (*ζ*) potential analysis of Bt-Ag_2_O NPs revealed that NPs synthesized have good stability and dispersity. There were no significant changes in *ζ* potential of Bt-Ag_2_O NPs after long time storage at − 20 °C; the *ζ* potential value of Bt-Ag_2_O NPs before and after storage was − 20.38 and − 16.80 mv, respectively. The *ζ* potential value is an important indicator of the stability of Ag_2_O NPs. It reveals the electrostatic repulsion between adjacent nanoparticles of similar charge in colloidal suspension, which is directly proportional to the agglomeration of nanoparticles and inversely proportional to the stability of nanoparticles (Elbahnasawy et al. 2021). pH was reported to be among the important factors that affect the zeta potential of synthesized nanoparticles, and the result corresponded to previous studies, which might be positive charge at lower pH and negative charge at higher pH (Morrison and Sydney 2002). In addition, the biomolecules attached to NP surfaces could offer steric stability and prevent these particles from aggregating (Maheshwaran et al. 2020).

Compared with Ag_2_O NPs and Bt, Bt-Ag_2_O NPs showed higher insecticidal activity against *T. castaneum*. When *T. castaneum* adults were treated for 14 days, the obtained toxicity regression equations of Bt, Ag_2_O NPs, and Bt-Ag_2_O NPs were Bt, *y* = 3.294 *x* + 2.824, *p* < 0.05; Ag_2_O NPs, *y* = 4.083 *x* + 4.661, *p* < 0.05; and Bt-Ag_2_O NPs, *y* = 4.743 *x* + 5.804, *p* < 0.05, where *y* is the mortality (%) in probits and *x* is the log_10_ dose. Based on these equations, the LC_50_ values of BT, Ag_2_O NPs, and Bt-Ag_2_O NPs were 0.139%, 0.072%, and 0.06% on day 14, respectively (Table S2). The low pesticidal concentrations of biosynthesized Ag_2_O NPs might be attributed to their smaller nanosized particles; due to their size, these NPs could easily penetrate through the cellular membrane, mitochondria, or DNA, leading to the denaturation of proteins and nucleic acids (Shehabeldine et al. 2021). There are many reports on the pesticidal activities of Ag_2_O NPs, and we found evidence from other similar studies. Ag NPs enter mosquito tissues through microconidia and nuclei at small concentrations, and the alterations in the gills and liver of *Oreochromis niloticus* treated with ZnO NPs synthesized by sea cucumbers were dose dependent (Elbahnasawy et al. 2023). Ag NPs showed high insecticidal activity by generating reactive oxygen types, oxidative stress, protein unfolding, cell membrane disruption, and inflammation, resulting in insect death, while green synthesized Ag_2_O NPs had higher cytotoxicity (Karunagaran et al. 2017; Rouhani and Samih 2013). These nanoparticles could act as carriers for *B. thuringiensis* crystal proteins with higher toxicity and lower environmental pollution and safety risks (Adams and Barbante 2013; Agarwal et al. 2017).

The development of *T. castaneum* was inhibited by Bt-Ag_2_O NPs, Ag_2_O NPs, and Bt at higher concentrations; the pupation and emergence rates were significantly lower than those of the controls, and the newly emerged adult body size also decreased. For example, at a concentration of 0.1%, the emergence rates of *T. castaneum* treated with Bt-Ag_2_O NPs, Ag_2_O NPs, and Bt were 2.22%, 6.83%, and 45.74%, respectively. These results were similar to those of previous studies; for example, Ag NPs significantly decreased *Drosophila melanogaster* fecundity, larval weight, rate of pupation, and emergence in a dose-dependent manner (Wang et al. 2023). Bt-Ag_2_O NPs showed higher pesticidal activity than Ag_2_O NPs and Bt, revealing the effect of bioactive substances from Bt against insects. Some insect pests display resistance to *Bt*, while metallic nanoparticles synthesized using *Bt* can strengthen its pesticidal activities, which can cause greater inhibition of α-amylase; as a result, the development of organisms or cells is slower and the body sizes are smaller (Wang et al. 2023). The details underlying the insect response to Ag/Ag_2_O NPs still need to be further studied in the future.

Ag_2_O NPs have potential applications as antimicrobial agents against drug-resistant bacteria, water disinfectants, and human pathogens (Dharmaraj et al. 2021; Yoo et al. 2019). In this paper, Bt-Ag_2_O NPs also showed excellent antifungal activities against *A. flavus* and *P. chrysogenum*, corresponding to previous similar studies on Ag_2_O NPs synthesized by *Bacillus paramycoides* against *Vibrio parahaemolyticus*, *Salmonella* sp., *Enterobacter* sp., and *Micrococcus* sp. (Dharmaraj et al. 2021), Ag_2_O NPs synthesized by *Staphylococcus epidermidis* against *S. epidermidis*, *S. aureus*, *A. fumigatus*, and *A. aureus* (Rashmi et al. 2020), and Ag_2_O/Ag NPs synthesized by *Streptomyces* sp. VITSTK7 against *A. fumigatus*, *A. niger*, and *A. flavus* (Thenmozhi et al. 2013). These Ag_2_O NPs were synthesized using microbes and significantly inhibited the growth and production of pathogens, and their antibacterial and antifungal activities were dose dependent. Silver ions are well known for their antimicrobial properties, which deactivate cellular enzymes and DNA, damage cell walls and membranes, and result in cell apoptosis. Ag_2_O NPs show higher cellular toxicity properties, and more silver ions are released from Ag_2_O NPs than from Ag NPs because the latter NPs are more stable. At the same time, all the NPs can function as bioactive ion reservoirs, leading to high antibacterial activity for a long period of time (Altaf et al. 2021; Bapat et al. 2022; Ou et al. 2015; Raja et al. 2017). It has reported that Ag_2_O NP-treated microbe (i.e., *S. aureus* and *E. coli*) exhibited changes in cell shape, such as cell wall breakdown, cell membrane lysis, rupture of cell structure, and leakage of intracellular materials (Yoo et al. 2019; Elbahnasawy et al. 2021). Ag_2_O NPs can perform antibacterial functions through other mechanisms, such as enzyme inactivation, genotoxic action, and photocatalytic action (Gudkov et al. 2023). As previously suggested, the higher antibacterial activity of NPs mostly results from contact with the cell wall rather than the NPs entering the cell; thus, the NPs are effective antibacterial agents for preventing the development of resistance (Dharmaraj et al. 2021).

In addition, the results revealed that the commercial Ag_2_O NPs showed stronger antifungal activities than the biosynthesized Bt-Ag_2_O NPs. This might occur because Ag plays a key role in antifungal activity and its antifungal activities were dose dependent; the content of Ag ions in Bt-Ag_2_O NPs was lower than that in commercial Ag_2_O NPs at the same dose used in the experiment (Hashem et al. 2022). Several other factors, such as chemical-physical properties, microbial species, treatment time, and environmental conditions, influence the antifungal activities of NPs synthesized by microbes (Danish et al. 2022; Wang et al. 2017). Ag_2_O NPs synthesized using Bt were effective at low doses with low toxicity and high specificity to achieve the desired result, so they might be a promising antifungal agent and pesticide (Danish et al. 2022).

In this paper, an eco-friendly and cost-effective method was used to produce Ag_2_O NPs using *B. thuringiensis*. The biosynthesized Bt-Ag_2_O NPs were characterized by UV‒vis, UV, XRD, SEM, HR-TEM, zeta potential, and ICP. Bt-Ag_2_O NPs showed a highly crystalline structure, small particles (18.24 nm), negative charge and moderate dispersibility and good stability. Based on FTIR results, Bt-Ag_2_O NPs are capped and stabilized with various biologically active ingredients from *B. thuringiensis.* The Bt-Ag_2_O NPs showed excellent insecticidal activity against *T. castaneum*, which significantly inhibited the development of insect larvae and caused death in a dose-dependent manner. Moreover, Bt-Ag_2_O NPs exhibited a significant effect against two tested fungi (*P. chrysogenum* and *A. flavus*), and *A. flavus* was more sensitive than *P. chrysogenum*. These results further demonstrated that Bt-Ag_2_O NPs are highly effective against *T. castaneum*, *P. chrysogenum*, and *A. flavus* and can be used as effective biopesticides and fungicides during grain storage. In the future, the mechanism of Bt-Ag_2_O NPs against insects and fungi should be further studied, as well as its environmental safety. Furthermore, the application of Bt-Ag_2_O NPs might be used in the fields of saline-alkali soil improvement and plant pathogen biological control.

## Supplementary Information

Below is the link to the electronic supplementary material.

Supplementary file1 (PDF 274 KB)

## Data Availability

The data used to support the findings of this study are available from the corresponding author upon request.
