# Supplementary material for: Biosynthesis of Bt-Ag2O nanoparticles using Bacillus thuringiensis and their pesticidal and antimicrobial activities
Source: Appl Microbiol Biotechnol. 2024 Jan 22;108:157. doi: 10.1007/s00253-023-12859-9 (PMC10803387; doi:10.1007/s00253-023-12859-9)
Supplement: Supplementary file 1 [file 253_2023_12859_MOESM1_ESM.pdf]

Title Page

- Journal name

Applied Microbiology and Biotechnology

- Manuscript Title

Biosynthesis of Bt-Ag_2O nanoparticles using *Bacillus thuringiensis* and their pesticidal and antimicrobial activities

- The name(s) of the author(s)

Jiajia Ge, Jianzhong Hu, Sufen.Cui*, Yirong Wang, CaiJiayi Xu, Wenzhuo Liu

1 Jiajia Ge and Jianzhong Hu share first authorship
*Author for correspondence; E-mail: Sufen18@just.edu.cn

- The affiliation(s) and address(es) of the author(s)

School of Grain Science and Technology, 212100, Jiangsu University of Science and Technology, Zhenjiang, China

- The e-mail address, telephone and fax numbers of the corresponding author

E-mail: Sufen18@just.edu.cn； +86 15000257686； +86 0511 85626711

Supporting information

Biosynthesis of Bt-Ag_2O nanoparticles using *Bacillus thuringiensis* and their pesticidal and antimicrobial activities

Jiajia Ge, Jianzhong Hu, Sufen.Cui*, Yirong Wang, CaiJiayi Xu, Wenzhuo Liu

School of Grain Science and Technology, 212100, Jiangsu University of Science and Technology, Zhenjiang, China

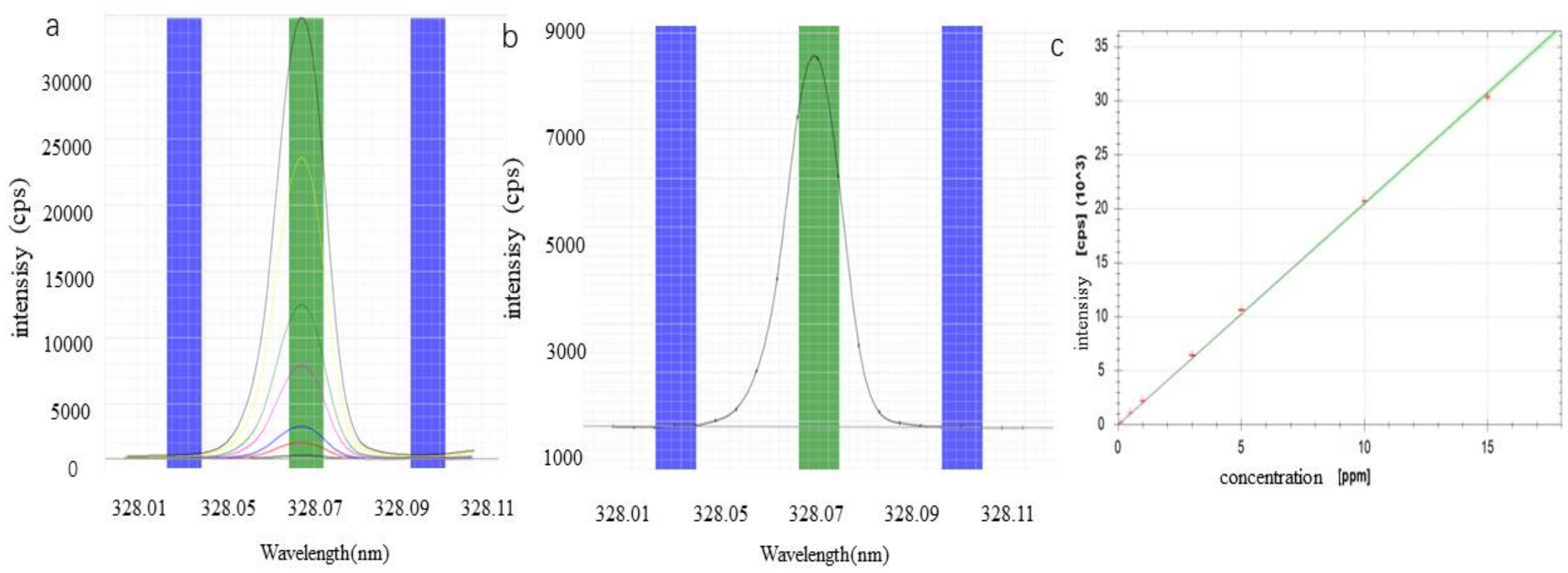

Fig. S1 ICP analysis of synthesized Bt-Ag_2O NPs

Table S1 The amount of Ag in the synthesized Bt-Ag_2O NPs determined by ICP-OES

Sample	M_0(g)	V_0 (mL)	test elements	C_o (mg/L)	f	C_1 (mg/L)	C_x (mg/kg)	W (%)
Bt-Ag_2O NPs	0.0235	25	Ag	3.368	50	168.400	179148.94	17.9

Note: M_0 is sample quality(g);C_x is the concentration of the analyzed ion(mg/kg),c_0 is the sample quality(g),f is the dilution rate, V_0 is the final solution after clear up;W is the amount of the analyzed ion(%)

Table S2 The LC_{50} of Bt ,Ag_2O NPs and Bt-Ag_2O NPs against *T. castaneum* adults for 14 days

**Pesticide**	Con(mg/kg flour)	14 days Mortality (%)±SD	**Slope function(S)**	**LC_{50}±SE (%)**	**LCL- UCL (mg/kg)**	**χ2**
Bt	Control	2.22±3.14a	Y=3.294x+2.824	0.139	0.101-0.602	22.53*
	0.2	2.22±3.14a				
	0.4	4.44±3.14a				
	0.6	17.78±3.14a				
	0.8	37.78±8.31b				
	1.0	43.17±8.17b				
Ag_2O NPs	Control	2.22±3.14a	Y=4.083x+4.661	0.072	0.62-0.087	19.659*
	0.2	4.31±3.05a				
	0.4	11.67±5.93a				

	0.6	31.11±11.33b				
	0.8	48.89±8.31b				
	1.0	83.65±3.61c				
Bt-Ag_2O NPs	Control	2.22±3.14a	Y=4.743x+5.804	0.06	0.051-0.070	24.041*
	0.2	4.44±3.14a				
	0.4	17.78±6.28a				
	0.6	44.44±8.31b				
	0.8	60.42±10.62b				
	1.0	100c				

Means followed by different letters are significantly different ($P < 0.05$; one-way ANOVA and Tukey's test). SD, standard deviation; SE, standard error; Con, concentration.LCL, lower confidence limits, UCL, upper confidence limits, $\chi 2$, Chi-square test.
